# Changes in T Lymphocyte Subsets in Different Tumors Before and After Radiotherapy: A Meta-analysis

**DOI:** 10.3389/fimmu.2021.648652

**Published:** 2021-06-16

**Authors:** Qin Wang, Shangbiao Li, Simiao Qiao, Zhihao Zheng, Xiaotong Duan, Xiaoxia Zhu

**Affiliations:** Department of Radiation Oncology, Zhujiang Hospital, Southern Medical University, Guangzhou, China

**Keywords:** meta-analysis, radiotherapy, T lymphocyte subsets, cellular immune, tumor types

## Abstract

**Purpose:**

Radiation therapy (RT) induces an immune response, but the relationship of this response with tumor type is not fully understood. This meta-analysis further elucidated this relationship by analyzing the changes in T lymphocyte subsets in different tumors before and after radiotherapy.

**Methods:**

We searched English-language electronic databases including PubMed, EMBASE, and the Cochrane Library to collect studies on the changes in peripheral blood CD3+ T lymphocytes, CD4+ T lymphocytes, and CD8+ T lymphocytes before and after radiotherapy in tumor patients from January 2015 to April 2021. The quality of the included literature was evaluated using the NOS scale provided by the Cochrane Collaboration, and statistical software RevMan 5.4 was used to analyze the included literature. P<0.05 was considered to indicate statistical significance.

**Results:**

A total of 19 studies in 16 articles involving 877 tumor patients were included. All data were collected within 1 month before or after radiotherapy. Meta-analysis showed that numbers of CD3+ T lymphocytes (SMD: -0.40; 95% CI [-0.75, -0.04]; p = 0.03) and CD4+ T lymphocytes (SMD: -0.43; 95% CI: [-0.85, -0.02]; p = 0.04) were significantly reduced after radiotherapy compared with before treatment, but there was no statistically significant difference for CD8+ T lymphocytes (SMD: 0.33; 95% CI: [-0.88, 0.74]; p = 0.12). Subgroup analysis showed that peripheral blood T lymphocytes decreased in head and neck cancer. However, in prostate cancer and breast cancer, there was no significant change in peripheral blood. 1 month after radiotherapy, it has a potential proliferation and activation effect on lymphocytes in esophageal cancer and lung cancer. The results showed that CD8+T lymphocytes increased in peripheral blood after SBRT. Radiotherapy alone reduced CD3+ T lymphocyte numbers.

**Conclusions:**

Within 1 month of radiotherapy, patients have obvious immunological changes, which can cause apoptosis and reduction of T lymphocytes, and affect the balance of peripheral blood immune cells. The degree of immune response induced by radiotherapy differed between tumor types.

## Introduction

Radiotherapy is an important treatment modality in the management of tumors (1). Sixty percent of newly diagnosed tumor patients receive radiotherapy as a first-line treatment plan (2), and radiotherapy alone or combined with surgery, chemotherapy, or targeted therapy can improve the local control rate in patients with various tumors and prolong their overall survival (3). In contrast to chemotherapy, radiotherapy can be used to achieve local tumor control with relatively few systemic side effects (4). Radiation therapy can directly kill a primary tumor by inducing double-strand DNA damage (5, 6), single-strand breaks (7), mismatch repair, chromosome aberrations (8), etc. It can also trigger the release of pro-inflammatory mediators and increase numbers of tumor-infiltrating immune cells (9), thereby activating an immune response. Ideally, this would reverse the immunosuppressive state of the tumor microenvironment and restore the immunogenicity of the primary tumor (10). Local radiotherapy can also cause abscopal effects (10–13), that is irradiation of a local tumor may also reduce the size of tumors outside the target area. The mechanism underlying this effect involves the release of pro-inflammatory factors by immune cells, enhancing the sensitivity of tumor cells to the immune system and thus enhancing the body’s anti-tumor immunity (14). However, in addition to immune activation, radiotherapy can promote the upregulation of immunosuppressive molecules, resulting in immunosuppressive effects (15). For example, activation of the DNA damage repair pathway by radiation-induced DNA damage upregulates CTLA-4 and PD-L1 expression (16); this can complicate the immune status of patients receiving radiotherapy.

Arina et al. proposed that T cells within a tumor may vary according to the tumor type, with different types having different expression patterns of radiation-resistance genes (17). Preclinical studies by Blair et al. have demonstrated that in tumors with poor radioimmunogenicity, such as pancreatic cancer, inhibition of the release or activity of endogenous adjuvants after radiotherapy may limit the extent of tumor control after radiotherapy (15). This suggests that the radiotherapy-mediated immune response varies greatly among different tumor types. Lymphocytes have an immune recognition function and are important cell components in the immune response. According to their phenotype and biological function, lymphocytes can be divided into T lymphocytes, B lymphocytes, and natural killer cell. Cellular immunity plays an important part in the body’s anti-tumor immune response, and T lymphocyte subsets have a leading role in the cellular immune response. T lymphocytes can be further divided into CD4+ T lymphocytes (CD3+ CD4+) and CD8+ T lymphocytes (CD3+ CD8+) according to their surface markers. CD4+ T lymphocytes are helper T cells, and CD8+ T lymphocytes are cytotoxic T cells.

The recent discovery of radiotherapy’s anticancer effects on the immune system has led to the recognition of radiotherapy’s ability to sensitize the tumor microenvironment to immunotherapy. However, the lack of understanding of the effect of radiotherapy on intratumoral immune balance hinders the optimization design of the combined radiotherapy and immune trial. A better understanding of how different types of tumor microenvironment influence the immune response after radiotherapy is needed. However, direct clinical studies of systemic immune responses to radiotherapy in patients with different tumor types have been lacking due to the wide variation in baseline among tumor patients. As a result, available research to explain it is rare. Furthermore, monitoring the level of lymphocyte subsets is important in guiding combination therapy for multiple cancers, making it possible to correlate immune status with treatment response in cancer patients receiving radiation therapy. Therefore, we conducted a systematic search and analysis of the existing literature in order to deepen our understanding of the systemic immune response to radiotherapy and to provide a theory for the differences in response to treatment among different tumor types.

## Methods

### Search Strategy and Selection Criteria

The PubMed, Cochrane, and Embase electronic databases were searched for records from January 2015 to April 2021, and the retrieved documents were screened and evaluated according to the inclusion criteria. Documents irrelevant to the research were excluded, and the screening was supplemented by a manual search for references in the included literature. The database search term was: (“radiotherapy” OR “irradiation” OR “radiation” OR “SABR” OR “SBRT”) AND (“immun*” OR “CD3+T” OR “CD4+T” OR “CD8+T” OR “PD-1” OR “PD-L1” OR “CTLA-4” OR “Tregs” OR “MDSC” OR “DC” OR “cytokines”). We restricted our searches to reports published in English.

The search strategy was developed in cooperation with an information expert. The inclusion criteria were as follows. (I) Research objects: Patients with cancer who have a clear pathological diagnosis. (II) Intervention measures: radiotherapy. (III) Outcome indicators: immune cell data (e.g. mean ± standard deviation) obtained before and after radiotherapy in peripheral blood, including data for CD3+ T lymphocytes, CD4+ T lymphocytes, and CD8+ T lymphocytes at baseline and after radiotherapy (within 1 month). (IV) Language restrictions: English. The exclusion criteria were as follows: (I) If there are duplicates, ambiguities, or publications that report on the same research population, only the latest, relevant and/or comprehensive publications will be included in the analysis, and others will be excluded. (II) Incomplete data or unavailable full-text literature. (III) These article types are excluded (i.e., case report, editorial). (IV) Outcome indicators: Not reporting outcomes of Interest or the immune cells are obtained from tumor infiltrating samples, not from peripheral blood. (V) Data obtained that could not distinguish between radiotherapy induced effects were excluded, such as those obtained when radiotherapy was combined with other concurrent treatments. (VI) Exclude tumor type or primary tumor disease that cannot be defined. (VII) Peripheral blood samples taken after radiotherapy were excluded over 1 month after the end of radiotherapy. (VIII) Studies with fewer than 10 participants were excluded. Articles that met the inclusion criteria in the initial screening by title and abstract were further screened based on their full text.

### Data Extraction and Study Quality

According to the PRISMA 2020 standard evaluation process (**Supplementary Material S1**), each document was screened by two people independently reading the title and abstract, after which another two reviewers read the full text. When two reviewers disagreed, a third person judged. The literature screening process was strictly controlled to ensure the quality of the research. The main information extracted in this study was: the first author of the study, publication date, sample size, gender and age of the research subject, study time of the intended population, country, tumor type, radiotherapy technology and dose, treatment method during radiotherapy, CD3+ T lymphocyte, CD4+ T lymphocyte, and CD8+ T lymphocyte count or percentage. The quality of included articles was evaluated by a modification of the Newcastle-Ottawa Scale (NOS) provided by the Cochrane Collaboration.

### Statistical Analysis

Statistical analysis was performed on the included literature with statistical software RevMan 5.4. The results of the literature included in this study were presented as continuous variable data with effect size given as standard mean difference (SMD) with 95% CI. Heterogeneity was measured using the I^2^ test. The detection of publication bias used funnel chart analysis. Data for CD3+, CD4+, and CD8+ T lymphocytes at baseline before radiotherapy and after radiotherapy were statistically analyzed. In addition, we conducted subgroup analysis according to tumor type, radiotherapy mode, and treatment method during radiotherapy. P<0.05 was considered statistically significant.

## Results

### Literature Search Results, Characteristics, and Quality of Included Studies

**Figure 1:**
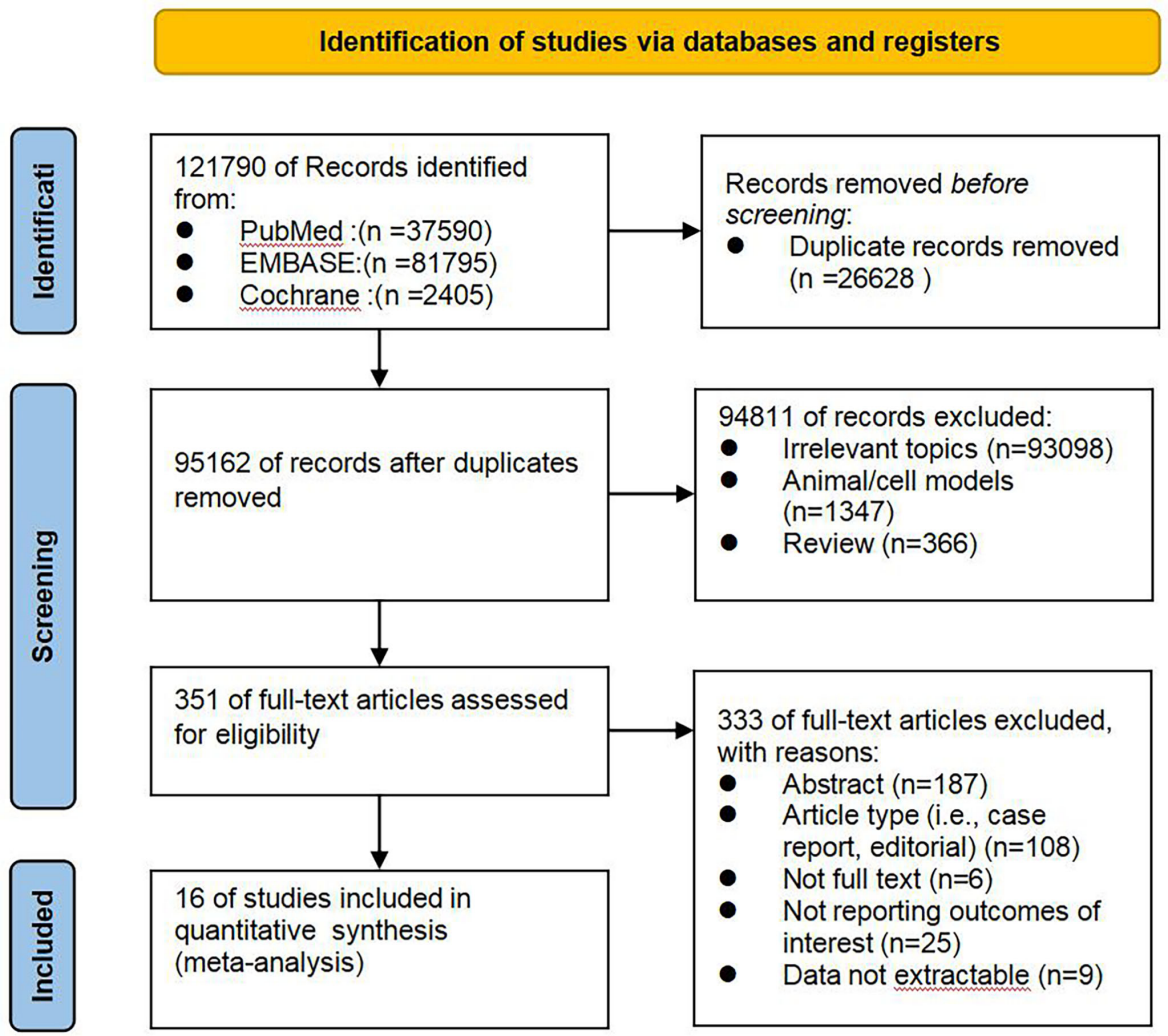
Flow diagram of studies identification and selection.

As shown in **Figure 1**, documents published from January 2015 to April 2021 were selected according to the search strategy: 121790 articles were initially identified, 26628 duplicates were excluded, 94811 articles were excluded based on their titles and abstracts, and 333 articles were excluded after careful reading of their full text carefully. Among them, 187 abstracts, and 108 articles do not match the type (i.e., case report, editorial), 6 articles for which the full text could not be found, and results of interest were not reported in 25 articles, data could not be extracted in 9 articles. Finally 19 studies in 16 articles were included in the meta-analysis, involving a total of 877 cases treated with radiotherapy. The sample size of cancer patients, ranged from 10 to 89. The countries the studies were performed in included the United States, Germany, Poland, China, Thailand, Spain. The radiotherapy technology were conventional radiotherapy (con-RT), stereotactic body radiotherapy (SBRT), Intensity-modulated radiation therapy(IMRT), and three-dimensional conformal radiotherapy (3D-CR). Treatment modes included radiotherapy alone and combined radiotherapy and chemotherapy (CCRT). Immune cell analysis was performed on blood samples before and after treatment. The basic characteristics of the included literature and the results of the quality evaluation are shown in **Table 1**. In exploring the changes of lymphocytes in each tumor after radiotherapy, and based on the clinical characteristics and heterogeneity of the studies included in **Table 1**, we finally adopted the random effects model for data analysis.

**Table 1:** Characteristics of the articles included in the meta-analysis.

Author	Year	Sampe size	Sex (F/M)	Age (years)	Country	Inclusion period	RT technology & dose	Cancer type	NOS score
Zhuang et al. (18)	2019	78	14/64	59.4 mean	China	2013-2017	SBRT 48-60Gy/5-10F	Liver Cancer	3
Zheng et al. (19)	2018	62	25/37	74 median	China	2012-2017	SBRT 48Gy/4F,60Gy/8F	Lung Cancer	5
Zhang et al. (20)	2018	48	19/29	57.4 mean	China	2015-2016	con-RT 50Gy/25F	Colorectal Cancer	5
Zhang2 et al. (20)	2018	28	14/14	59.5 mean	China	2015-2016	3D-CRT 45.0-50.4Gy/23-28F	Colorectal Cancer	5
Zhang3 et al. (20)	2018	17	3/14	63.1 mean	China	2015-2016	IMRT 45.0-50.4Gy/23-28F	Colorectal Cancer	5
Yuan et al. (21)	2018	30	30/0	48 mean	China	2015-2016	con-RT 50Gy/25F	Breast Cancer	4
Luo et al. (22)	2018	82	31/51	NK	China	2016-2017	con-RT 50-66Gy/20-33F	Lung Cancer	3
Dovsak et al. (23)	2018	56	12/44	62 mean	United States	2008-2013	con-RT 60-66Gy/28-54F	Head and Neck Cancer	5
Sage et al. (24)	2017	10	NK	NK	Germany	NK	con-RT 70-74Gy/35-37F	Prostate Cancer	3
Rutkowski et al. (25)	2017	89	29/60	74 median	Poland	NK	SBRT 54-60Gy/3-8F	Lung Cancer	6
E. K. et al. (26)	2016	40	40/0	NK	Germany	NK	con-RT 60Gy/30F	Lung Cancer	5
Sangthawan et al. (27)	2015	36	5/31	60	Thailand	2006-2007	con-RT 60Gy/30F	Head and Neck Cance	4
Chen et al. (28)	2019	37	12/25	53 median	China	2015-2016	IMRT NK	Head and Neck Cancer	4
Balazs et al. (29)	2019	23	9/14	64 median	Poland	2016-2016	IMRT 51-74Gy/17-46F	Head and Neck Cancer	3
Wild et al. (30)	2016	32	13/19	67 median	United States	NK	SBRT 33Gy/5F	Pancreatic Cancer	4
Linares et al. (31)	2021	13	13/0	66 median	Spain	2016-2018	IORT 20Gyx1F	Breast Cancer	4
Lv et al. (32)	2020	31	6/25	66 mean	China	NK	IMRT 45-60 Gy,1.8–2.0 Gy/F	esophageal cancer	5
Da1 (33)	2020	88	43/45	63 median	United States	2013-2018	con-RT 45 Gy/15F	Lung Cancer	6
Da2 (33)	2020	77	50/27	66 median	United States	2013-2018	SBRT 50 Gy/4F,60 Gy/10F	Lung Cancer	6

NK, unknown since not mentioned in the literature.

### Conventional Pairwise Meta-Analysis

**Figure 2:**
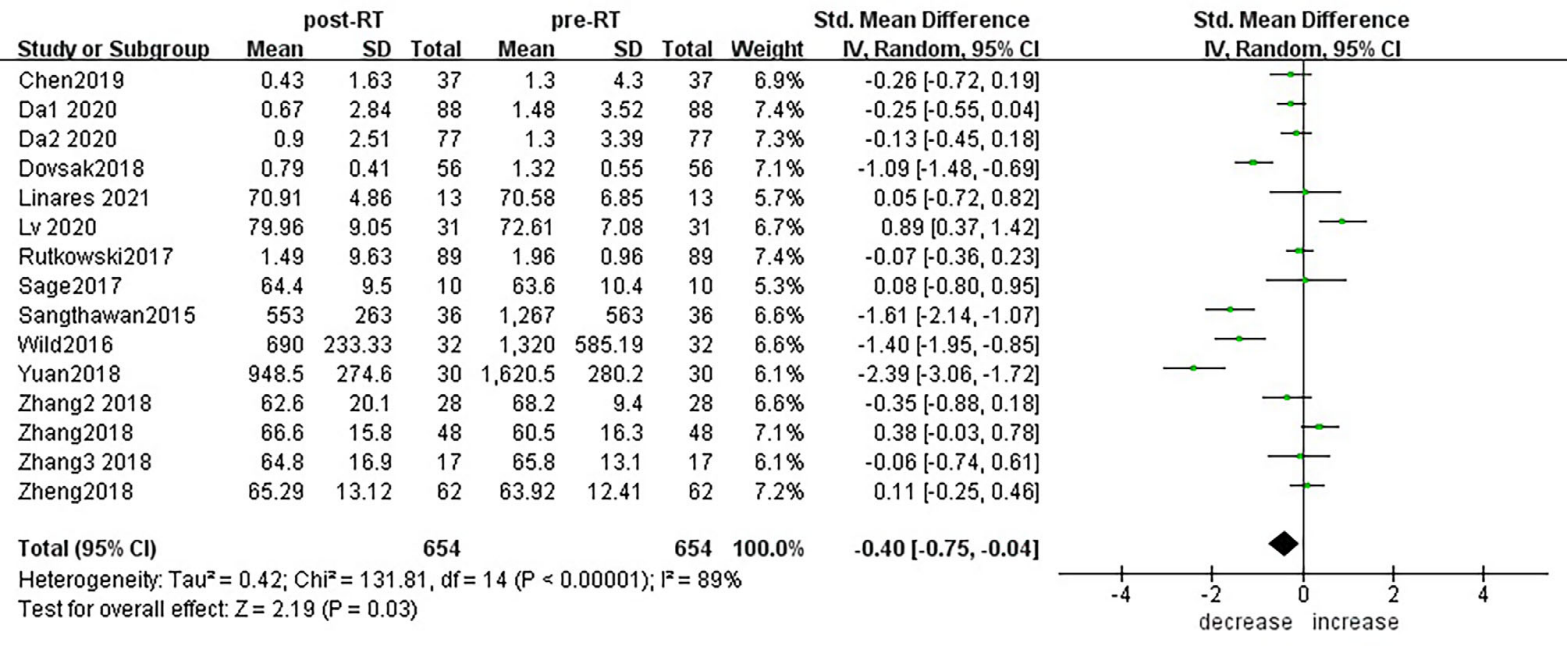
Forest plot for changes of CD3+T lymphocytes after radiotherapy compared with before radiotherapy. decrease: CD3+T lymphocytes were less after radiotherapy than before. increase: CD3+T lymphocytes were increased after radiotherapy than before. Data are presented as standard mean difference [SMD] and 95% CI.

**Figure 3:**
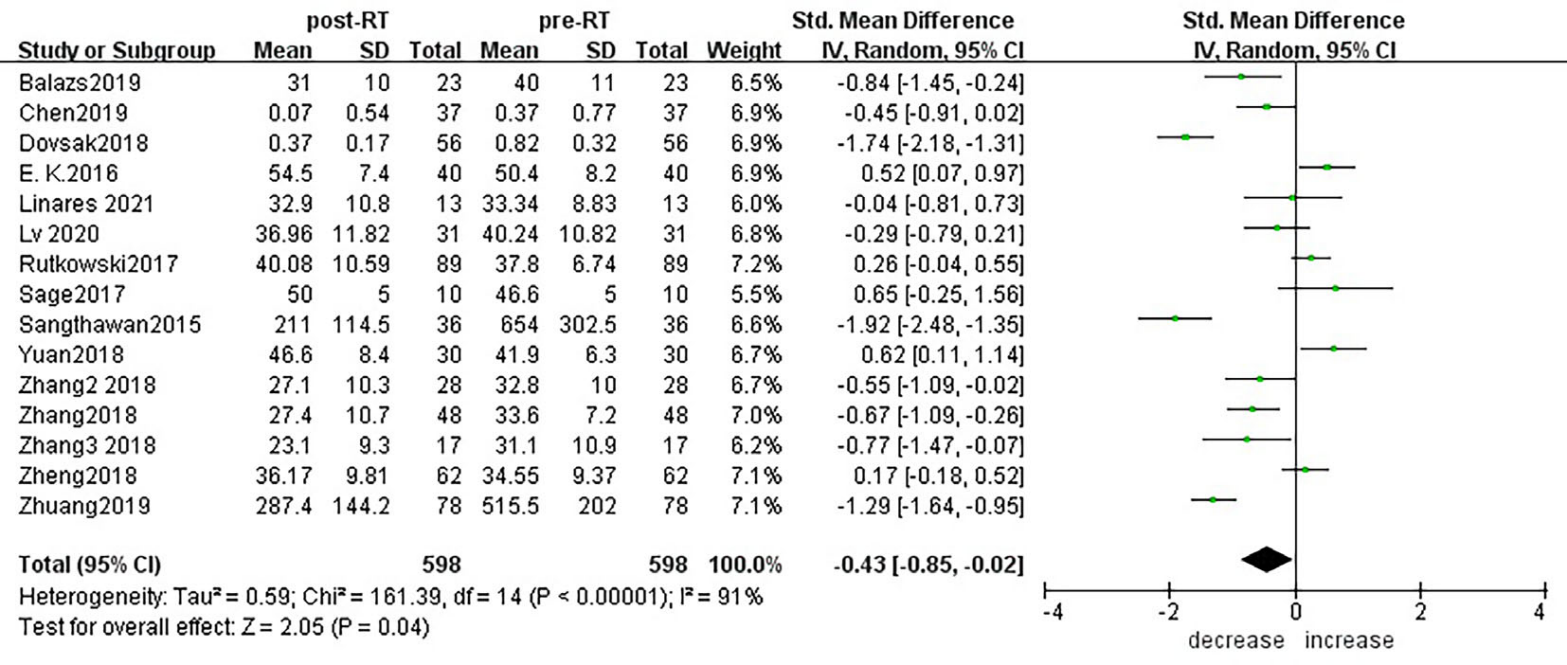
Forest plot for changes of CD4+T lymphocytes after radiotherapy compared with before radiotherapy. decrease: CD4+T lymphocytes were less after radiotherapy than before. increase: CD4+T lymphocytes were increased after radiotherapy than before.

**Figure 4:**
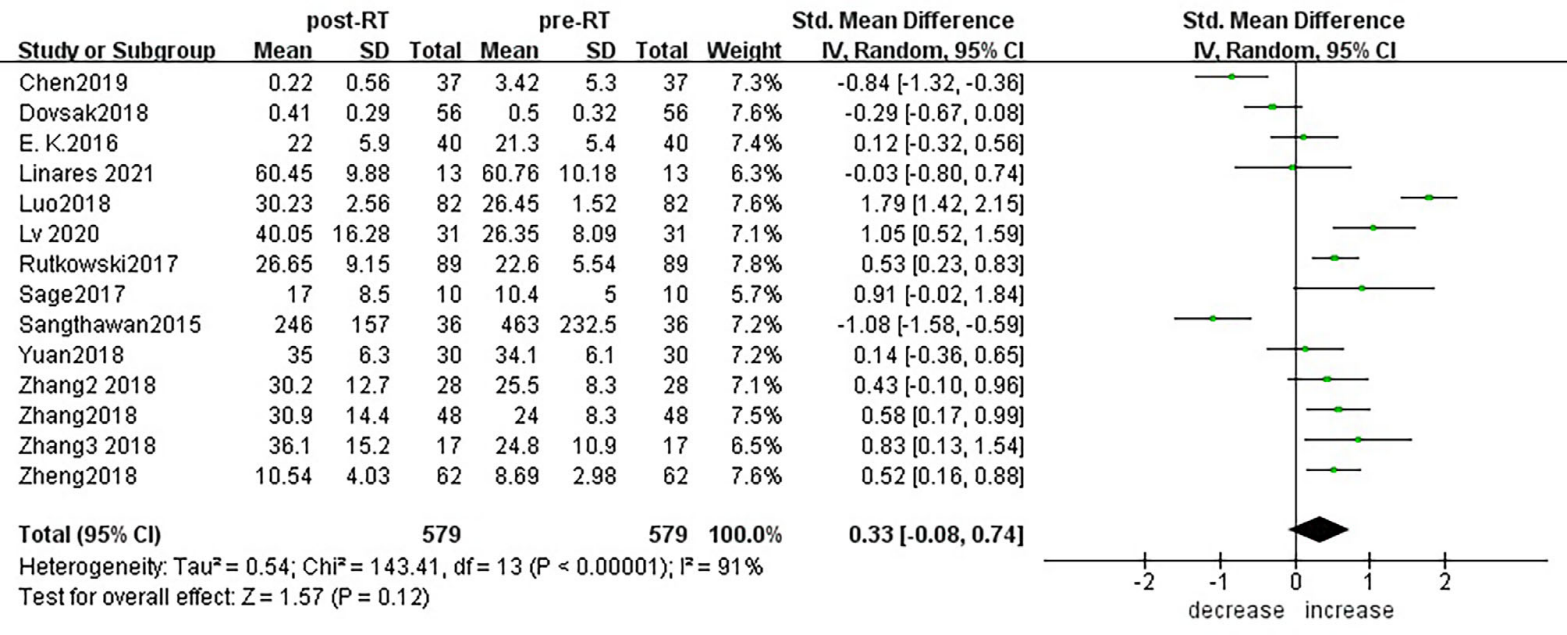
Forest plot for changes of CD8+T lymphocytes after radiotherapy compared with before radiotherapy. decrease: CD8+T lymphocytes were less after radiotherapy than before. increase: CD8+T lymphocytes were increased after radiotherapy than before.

According to sensitivity analysis and bias analysis (**Supplementary Materials S2, 3**), 14 studies from 1^2^ articles were finally included to analyze the changes of CD3+T lymphocytes after radiotherapy. The heterogeneity test indicated that there was heterogeneity among the studies (I^2^ = 89%, p<0.05). The combined results showed a statistically significant decrease in CD3+ T lymphocyte (SMD: -0.40; 95% CI [-0.75, -0.04]; p=0.03) after radiotherapy compared with baseline (**Figure 2**). Then we explored the changes in CD4+ T lymphocyte after radiotherapy from 15 studies based on sensitivity analysis sensitivity analysis and bias analysis (**Supplementary Materials S4, 5**). The heterogeneity test indicated that there was heterogeneity among the studies (I^2^ = 91%, p<0.05). Similarly, CD4+ T lymphocytes in peripheral blood also decreased after radiotherapy compared with baseline (SMD: -0.43; 95% CI: [-0.85, -0.02]; p=0.04) (**Figure 3**). According to sensitivity analysis and bias analysis (**Supplementary Materials S6, 7**), however, in 14 included studies showed no significant difference in CD8+ T lymphocyte before and after treatment (SMD: 0.33; 95% CI: [-0.88, 0.74]; p=0.12) (**Figure 4**), although there is an increasing trend. The heterogeneity test indicated that there was heterogeneity among the studies (I^2^ = 91%, p<0.05).

Within 1 month of radiotherapy, patients had significant immunological changes that could cause T lymphocyte apoptosis and decrease, affecting the balance of peripheral blood immune cells.

### Subgroup Analysis

**Figure 5:**
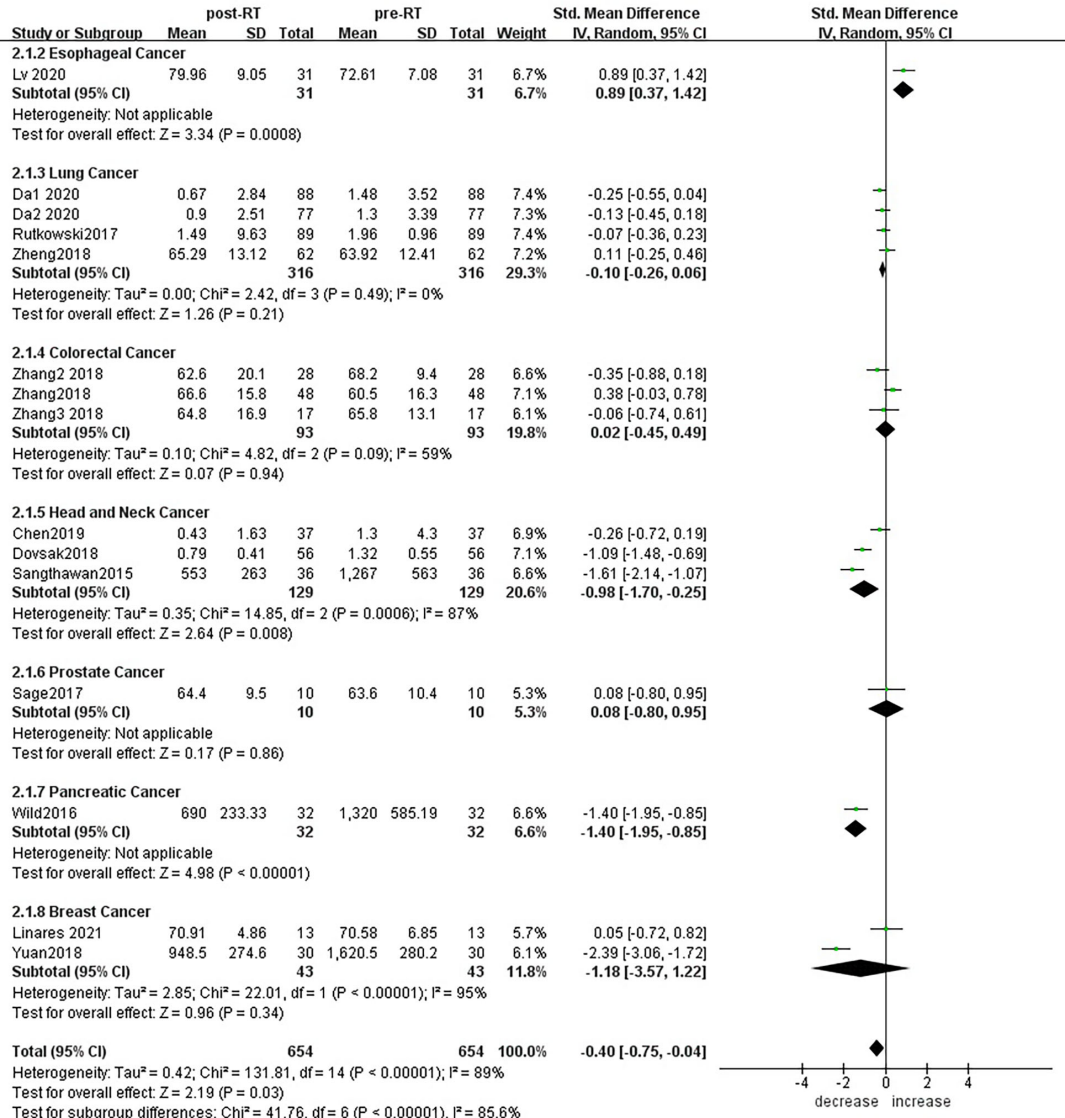
Forest plots of subgroup analysis for changes of CD3+ T lymphocyte after radiotherapy compared with before radiotherapy in different tumors.

**Figure 6:**
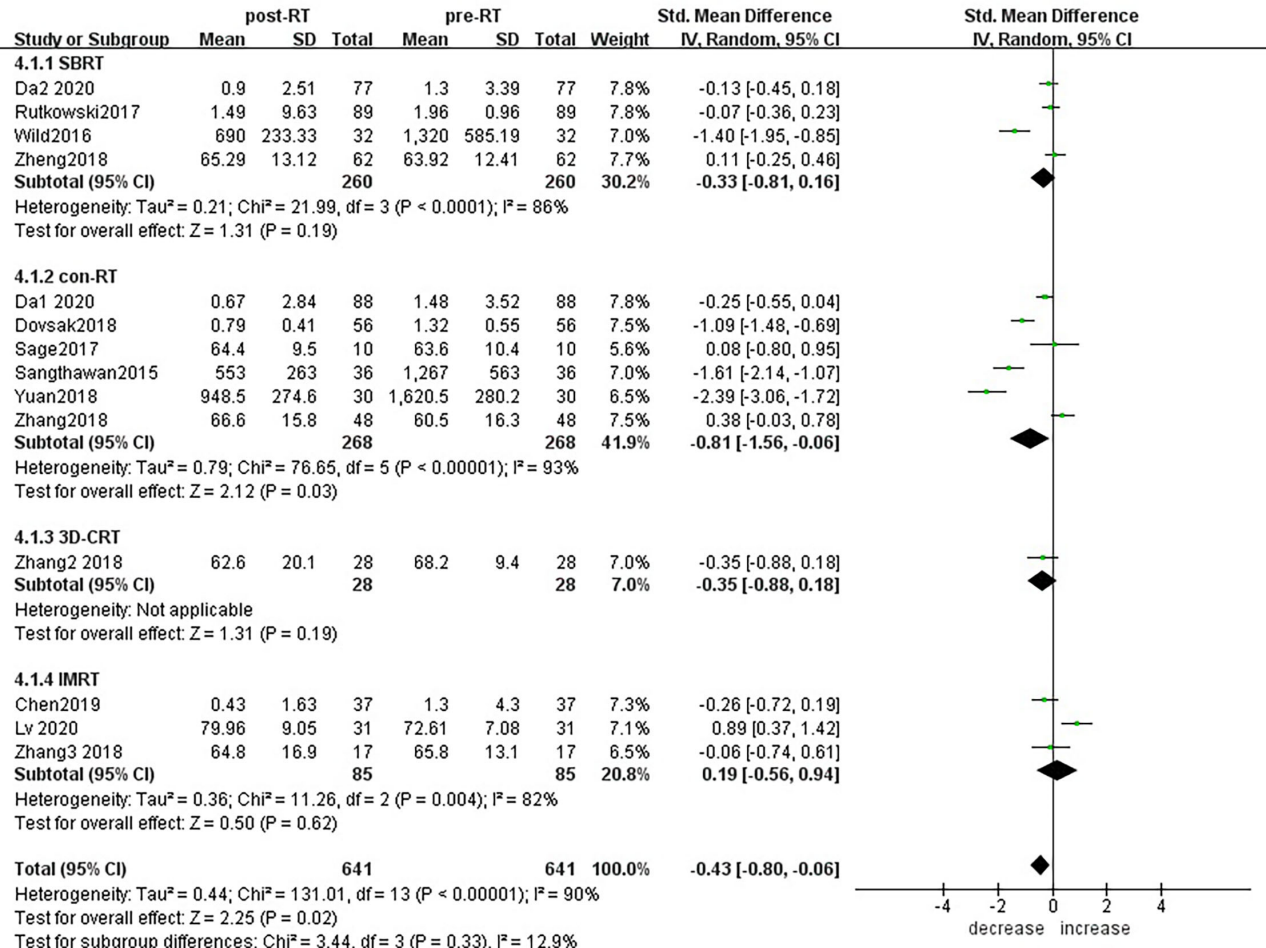
Forest plots of subgroup analysis for changes of CD3+ T lymphocyte after radiotherapy compared with before radiotherapy with different radiotherapy technology. con-RT, stereotactic body radiotherapy; SBRT, stereotactic radiotherapy; IMRT, intensity-modulated radiotherapy; 3D-CRT, three-dimensional conformal radiotherapy.

**Figure 7:**
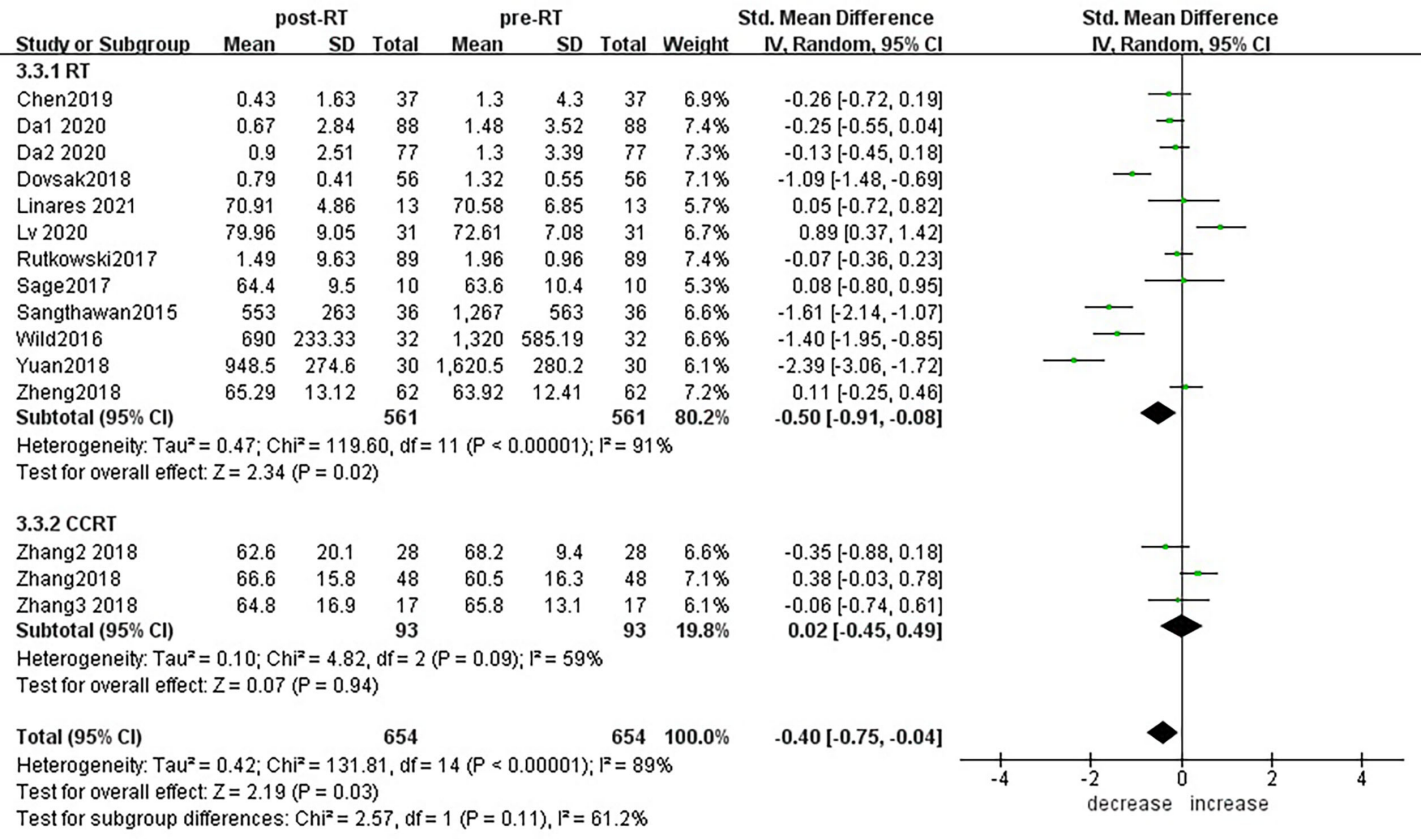
Forest plots of subgroup analysis for changes of CD3+ T lymphocyte after radiotherapy compared with before radiotherapy in different treatment modes.

Subgroup analysis of the changes in CD3+ T lymphocytes before and after radiotherapy was performed. A total of 15 studies of different primary tumor types were included (**Figure 5**), in which statistically significant decreases in numbers of CD3+ T lymphocytes were found in head and neck cancer (SMD:-0.98; 95% CI [-1.70, -0.25]; p<0.01) and pancreatic cancer (SMD: -1.40; 95% CI [-1.95, -0.85]; p<0.01). However, in esophageal cancer (SMD:0.89; 95% CI [0.37, 1.42]; p<0.01),CD3+ T lymphocytes increased after radiotherapy. ln lung cancer (SMD: -0. 10; 95% CI [-0.26,0.06]; p=0.21), colorectal cancer (SMD: 0.02; 95% CI [-0.45, 0.49]; p=0.94), prostate cancer (SMD: 0.08; 95% CI [-0.80, 0.95]; p=0.17) and breast cancer (SMD: -1.18; 95% CI [-3.57, 1.22]; p=0.34) patients, there were no statistically significant changes in CD3+ T lymphocyte numbers after radiotherapy. With the improvement of radiotherapy technology, precision radiotherapy is gradually mature, which can effectively improve the survival rate of patients, and its significant clinical efficacy has been widely recognized. To further explore the effects of radiotherapy technology on CD3+ T lymphocytes, 14 studies were included for subgroup analysis (**Figure 6**). After conventional radiotherapy(con-RT), the numbers of CD3+ T lymphocytes decreased significantly compared with baseline (SMD: -0.81; 95% CI [-1.56, -0.06]; p=0.03); however, there was no significant change after SBRT (SMD: -0.33; 95% CI [-0.81, 0.16]; p=0.19), 3D-CRT (SMD: -0.35; 95% CI [-0.88,0.18]; p=0.19), or IMRT (SMD: 0.19; 95% CI [-0.56,0.94]; p=0.62). Next, we explored the effects of the treatment mode on CD3+ T lymphocytes during radiotherapy. A total of 15 studies were included (**Figure 7**). After radiotherapy alone, CD3+ T lymphocytes decreased significantly compared with baseline (SMD: -0.50; 95% CI [-0.91, -0.08]; p=0.02); however, there was no significant change after concurrent radiotherapy and chemotherapy (SMD: 0.02; 95% CI [-0.45, 0.49]; p=0.94).

**Figure 8:**
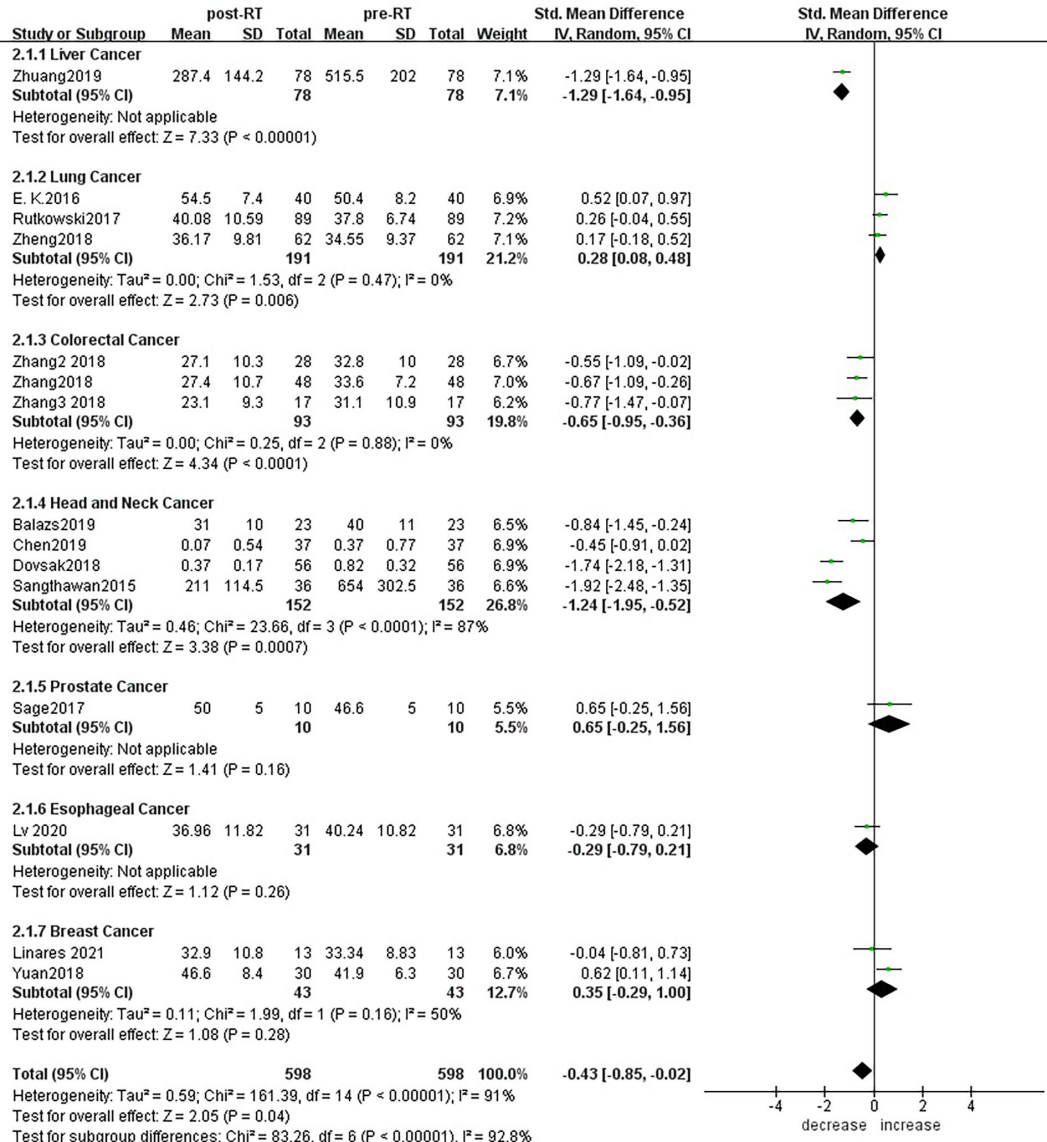
Forest plots of subgroup analysis for changes of CD4+ T lymphocyte after radiotherapy compared with before radiotherapy in different tumors.

For the subgroup analysis of CD4+ T lymphocytes before and after radiotherapy,15 studies were included (**Figure 8**). For primary tumors including liver cancer (SMD: -1.29; 95% CI [-1.64, -0.95]; p<0.01), colorectal cancer (SMD: -0.65; 95% CI [-0.95, -0.36]; p<0.01) and head and neck cancer (SMD: -1.24; 95% CI [-1.95, -0.52]; p< 0.01), CD4+ T lymphocyte numbers decreased after radiotherapy compared with baseline, and the differences were statistically significant.CD4+ T lymphocytes increased in patients with lung cancer (SMD: 0.28; 95% CI [0.08, 0.48]; p< 0.01) 1 month after radiotherapy. However, there was no significant difference in patients with prostate cancer (SMD: 0.65; 95% CI [-0.25, 1.56]; p=0.16), esophageal cancer (SMD: -0.29; 95% CI [-0.79, 0.21]; p=0.26) and breast cancer (SMD: 0.35; 95% CI [-0.29, 1.00]; p=0.28). To explore the effects of radiotherapy technology on CD4+ T lymphocytes, 14 studies were included in the subgroup analysis (**Supplementary Material S8**). There were no significant changes in CD4+ T lymphocyte numbers after conventional radiotherapy (SMD: -0.44; 95% CI [-1.37, 0.49]; p=0.35) or SBRT (SMD: -0.29; 95% CI [-1.26, 0.68]; p=0.56), but there were statistically significant decreases after 3D-CRT (SMD: -0.55; 95% CI [-1.09, -0.02]; p=0.04) and IMRT (SMD: -0.53; 95% CI [-0.80, -0.26]; p<0.01). Next, we considered the effects of treatment mode on CD4+ T lymphocytes during radiotherapy. A total of 15 studies were included (**Supplementary Material S9**). CD4+ T lymphocyte numbers remained essentially unchanged after radiotherapy alone (SMD: - 0.37; 95% CI [-0.88, 0.13]; p=0.15), but combined with chemotherapy and radiotherapy decreased, with statistically significant difference (SMD: -0.65; 95% CI [-0.95, -0.36]; p< 0.01).

**Figure 9:**
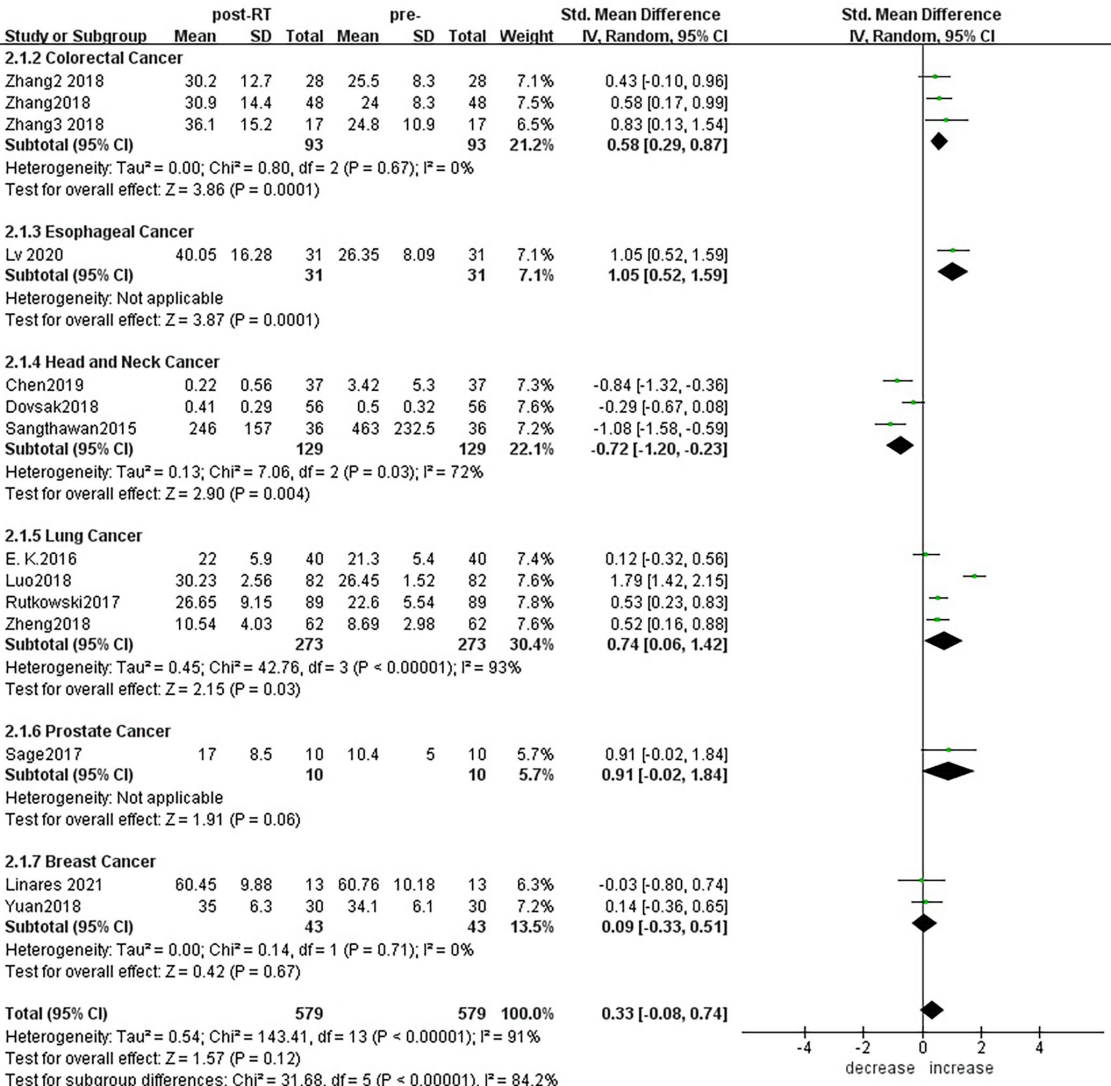
Forest plots of subgroup analysis for changes of CD8+ T lymphocyte after radiotherapy compared with before radiotherapy in different tumors.

For the subgroup analysis of CD8+ T lymphocytes before and after radiotherapy (**Figure 9**), a total of 14 studies were included. In terms of the primary tumor type, esophageal cancer (SMD:1.05;95% CI [0.52, 1.59]; p<0.01), colorectal cancer (SMD: 0.58; 95% CI [0.29, 0.87]; p<0.01), lung cancer (SMD: 0.74; 95% CI [0.06, 1.42]; p=0.03) showed statistically significant increases from baseline in CD8+ T lymphocyte numbers after radiotherapy; head and neck cancer (SMD: -0.72; 95% CI [-1.20, -0.23]; p=0.004) showed significant decreases; and there was no significant change for prostate cancer (SMD: 0.91; 95% CI [-0.02, 1.84]; p=0.06) or breast cancer (SMD: 0.09; 95% CI [-0.33, 0.51]; p=0.67). To explore the effects of radiotherapy technology on CD8+ T lymphocytes, 13 studies were included for subgroup analysis (**Supplementary Material S10**). After conventional radiotherapy (SMD: 0.30; 95% CI [-0.43, 1.03]; p=0.42) and IMRT (SMD: 0.34; 95% CI [-0.95, 1.62]; p=0.61) and 3D-CRT (SMD: 0.43; 95% CI [-0.10, 0.96]; p=0.11), CD8+ T lymphocyte numbers remained essentially unchanged with no statistically significant differences. But after SBRT, CD8+ T lymphocyte showed significant increased (SMD: 0.53; 95% CI [0.30, 0.76]; p< 0.01). Next, we explored the effect of the treatment mode on CD8+ T lymphocytes during radiotherapy. A total of 14 studies were included (**Supplementary Material S11**). CD8+ T lymphocyte numbers remained essentially unchanged after radiotherapy alone (SMD:0.08; 95% CI [-0.32, 0.49]; p=0.68) but increased compared with baseline after radiotherapy combined with chemotherapy, and the difference was statistically significant (SMD: 0.92; 95% CI [0.21, 1.63]; p=0.01).

Subgroup analysis of different cancer types showed that peripheral blood CD3+ T lymphocytes, CD4+ T lymphocytes and CD8+ T lymphocytes decreased after radiotherapy compared with baseline in head and neck cancer, indicating that within 1 month after radiotherapy, T lymphocytes in peripheral blood are damaged. However, in prostate cancer and breast cancer, there was no significant change in peripheral blood CD3+T, CD4+T and CD8+T lymphocytes after radiotherapy. Within 1 month after radiotherapy, the peripheral blood CD3+T and CD8+T lymphocytes of patients with esophageal cancer increased statistically compared with before, and there was no statistical difference in CD4+T lymphocytes. The peripheral blood CD4+ T and CD8+ T lymphocytes of patients with lung cancer increased compared with those before radiotherapy, and there was no statistical difference in CD3+ T lymphocytes. This suggests that 1 month after radiotherapy, it has a potential proliferation and activation effect on lymphocytes in esophageal cancer and lung cancer. However, further typing analysis of immune cell function is still needed to confirm our conclusion. In fact, many literatures have reported that the increase of peripheral blood lymphocytes is beneficial to survival and prognosis. In particular, CD8+ T lymphocytes play a key role in anti-tumor effects, and their degree of tumor infiltration is related to treatment response. Therefore, the changes in peripheral blood immune cells studied by this meta-analysis have certain significance for clinical treatment. From these data, we found differences in peripheral blood T lymphocytes among different tumor types after radiotherapy.

Clinically, radiotherapy technology and treatment modes have a certain influence on the therapeutic effect achieved; we performed a further subgroup analysis to investigate this. The results showed that CD8+T lymphocytes increased in peripheral blood after SBRT. Conventional fractionation radiotherapy, 3D-CRT and IMRT showed a tendency to decrease peripheral blood T lymphocytes. Radiotherapy alone reduced CD3+ T lymphocyte numbers.

## Discussion

The host immune responses involved in the progression of malignant tumors have been extensively studied, and related anti-cancer strategies have been developed, but the underlying mechanisms have not yet been fully elucidated (34–36). Treatment effects are different for different tumor types, and the mechanisms of radiation resistant tumors deserve further exploration. In order to solve these problems, clinical studies are being carried out to find effective biomarkers for predicting immune response in cancer patients receiving radiotherapy. Circulating T lymphocytes may be an indicator of the immune response that targets tumors and can serve as predictive biomarkers. In this way, they can serve as a valuable substitute for therapeutic response and also overcome the limitations of tissue-based biomarkers.

Immunosuppression is an unexpected result of radiotherapy, and the resulting immunosuppressive microenvironment is considered to be a side effect of radiotherapy. Interestingly, recent studies have found that treatment-induced lymphopenia is associated with poor prognosis in many cancers (34, 37, 38). Lymphopenia before treatment is an independent predictor of poor prognosis in muscular invasive bladder cancer and advanced bladder cancer (39). This may be a manifestation of tumor-induced immunosuppression and a driver of tumor progression. In advanced rectal cancer, the relative lymphocyte count before treatment has a significant impact on the pathological tumor response (tumor downstaging) of locally advanced rectal cancer after preoperative radiotherapy and chemotherapy (36). Enhancing the lymphocyte-mediated immune response can improve the effects of preoperative radiotherapy and chemotherapy for rectal cancer, suggesting that the peripheral blood lymphocyte count could be a potential strategy for patient stratification. From a radiobiological point of view, lymphocytes are extremely sensitive to radiation, and there is a concern that radiotherapy-related lymphopenia may affect the response to immunotherapy (40). Although the absolute peripheral blood lymphocyte count is the best-studied predictive biomarker in cancer patients receiving radiotherapy, reductions in other lymphocyte subsets after radiotherapy, especially CD4+ T cells, seem to be associated with survival outcomes (41, 42). These changes are closely related to the primary tumor type, the radiation technology (photon *vs*. proton or heavy ion therapy), the dose plan (low segmentation or conventional segmentation), and the time point of blood sampling (before, during, or after radiotherapy). In addition, an increase in the ratio of CD4+:CD8+ T lymphocytes has been shown to be related to an increase in the effective rate of prostate cancer patients after receiving carbon ion radiotherapy (43). By contrast, increased numbers of CD8+ T cells, a decreased ratio of CD4+:CD8+ T cells, and an increased ratio of activated CD8+ T cells and PD-1+CD8+ T cells to CD4+ T cells were found to be beneficial in a phase I study of patients with lung or liver metastases of any type of solid tumor undergoing SBRT combined with ipilimumab treatment (44). There are several explanations for these seemingly contradictory observations. The distribution of lymphocyte subtypes in the human body is not uniform; for example, B cells tend to accumulate in the spleen, and lymphocytes with immature antigens accumulate in lymph nodes (45). Therefore, certain lymphocyte subgroups are preferentially consumed. There may also be a dependence on the specific subgroup and the radiation sensitivity of the exposure site, although there have been no systematic studies on these effects. Current data indicate that B cells, as well as CD4+ T cells and perhaps naive T cells, are more radiosensitive than CD8+ T cells, memory T cells, and regulatory T cells (46–49). Even in patients treated in the same anatomical area, there have been different reports on the distribution of circulating lymphocytes in various tissues after radiotherapy. Immune differences among tissues and organs could also explain the differences in the immune responses of different tumors after radiotherapy. For example, clinical studies have reported that the degree of immune response caused by radiotherapy is related to the location of radiotherapy. Organs such as the skin, gastrointestinal tract, and lungs are partly connected to the outside environment; thus, their immune system is different from that of internal organs. Owing to long-term exposure to metabolites, the liver has also distinct immune responses. In addition, the central nervous system, and organs of the reproductive system (e.g., the testes) are special immune-exempt organs (50). In clinical studies, there are differences in patient stage, age, underlying disease, etc., as well as variations in the radiotherapy doses used, limiting direct comparison of immune changes in tumors after radiotherapy. However, Preclinical studies in minimizing these differences were found that when a series of tumors received the same dose of radiation therapy, the improved treatment response only depended on the presence of CD8+ T cells in radioimmunogenic tumors. That is, inherent radiation sensitivity was irrelevant (15). These findings are controversial, and further research is needed to explain the potential immune-related mechanisms underlying the responses of different tumor types to radiation.

In this meta-analysis, generally speaking, within 1 month of radiotherapy, patients have obvious immunological changes, which can cause apoptosis and reduction of T lymphocytes, and affect the balance of peripheral blood immune cells. Finally we found that CD3+ T lymphocytes and CD4+T lymphocytes decreased, CD8+T lymphocytes showed no statistical difference compared with those before radiotherapy.

Evaluating the level of T lymphocytes in peripheral blood may be important for guiding the treatment of patients with different tumors. Subgroup analysis of different cancer types showed that T lymphocytes decreased after radiotherapy in head and neck cancer. However, in prostate cancer and breast cancer, there was no significant change in peripheral blood T lymphocytes after radiotherapy. But it has a potential proliferation and activation effect on lymphocytes in esophageal cancer and lung cancer. However, further typing analysis of immune cell function is still needed to confirm our conclusion.

Changes in RT technology may reduce exposure to circulating blood. Previous studies have shown that reducing the number of fraction and/or narrowing the field of radiation can preserve circulating lymphocytes (30). Subgroup analysis of radiotherapy technology showed that CD8+ T lymphocytes increased after SBRT. Conventional fractionation radiotherapy, 3D-CRT and IMRT showed a tendency to decrease peripheral blood T lymphocytes. The correlation of dose-response to radiotherapy is currently unclear. One of the possible reasons is that lymphocytes are highly radiosensitive. Regarding RT technology, it is unclear whether SBRT is associated with improved response rate and prognosis due to enhanced lymphocyte preservation, increased antigen presentation, or both (33). But we suspect that part of the reason may be that SBRT reduces the target volume and the fractions so it can protect circulating lymphocytes.

In addition, as therapeutic agents are increasingly targeted and used in conjunction with the immune system, maintaining lymphocyte counts can potentially improve response to treatment (51). Subgroup analysis showed that radiotherapy alone reduced CD3+ T lymphocyte numbers. This also suggests that the role of RT alone is not enough to establish protective anti-tumor immunity, but it may be able to enhance the effects of other immunotherapies (51). In the era of radiation-induced immune regulation, maintaining immune function and cells is essential to maximize the efficacy of immune stimulating compounds (52).

Lymphopenia may be related to the poor prognosis of cancer patients receiving RT treatment (53). However, evaluating tumor infiltrating lymphocytes (TIL) will require tissue biopsies at consecutive time points, which are not easily available in tumor patients. Compared with tumor biopsy, peripheral blood has the advantages of minimally invasive and feasibility of continuous collection.

Immune function can predict the response of patients receiving immunoradiotherapy. In recent years, people have paid more and more attention to a combination of radiotherapy (RT) and immunotherapy as part of tumor treatment. Due to the sensitivity of circulating lymphocytes to radiation (necessary for anti-tumor immune response), RT is often accompanied by lymphopenia, which in turn will affect recurrence and survival (54, 55). Although the immune status before RT is an unchangeable predictor of PFS, the immune status after RT is changeable and is related to the improvement of PFS in the traditional RT cohort. Therefore, maintaining immune function and cells is essential to maximize the efficacy of immunostimulatory compounds. This requires achieving a balance between the immune system and inflammation in patients, which poses a challenge to clinical and scientific researchers. The immune system is not only regulated by radiation, but also responds to changes in the tumor microenvironment. Although radiation therapy has an immune-stimulating effect through the induction of neoantigens and immune-activated danger signals (56), it also has immunosuppressive effects, such as lymphopenia. However, the effect depends on the timing of the blood count and the timing of the lymphopenia. Some studies reported that these changes recovered within 2 months, 3 months, 6 months, and 1 year after treatment. Eckert et al. found that except Tregs, all subgroups showed increased proliferation rates during RT and returned to normal levels three months after the end of treatment (57). Different populations and lymphocyte subgroups have different immune cell recovery time, coupled with the influence of different radiotherapy techniques and fraction schemes, so current studies have not reached a consensus on when lymphocytes will start to recover after receiving radiation.

In fact, in the process of screening, there were 9 articles whose data could not be extracted and were excluded, among which 3 articles were qualitatively analyzed (**Supplementary Material S12**). Overall, the damage of radiotherapy to immune cells was consistent with the quantitative analysis results we conducted before. After the full article was screened, sensitivity analysis and bias analysis were conducted, and finally 4 studies were excluded in different subgroup analyses. The heterogeneity of the article was reduced to some extent. At the same time, our results have not changed, indicating that our final results are relatively stable.

There are some limitations to our study. First, most of the included studies used radiotherapy alone and collected peripheral blood within 1 month after radiotherapy, at which time the immunosuppressive effect of radiotherapy is predominant. As immune changes after radiotherapy will evolve over time, further research including long-term follow-up studies is needed to determine the best time point after radiotherapy at which to measure immune cells. In addition, the baseline characteristics of the included patients are critical: age, gender, tumor stage, histological type, treatment history, number of treatment lines, etc. can all have an important impact on test results. The studies we included focused on different tumors, which is inherently heterogeneous. However, there was also heterogeneity among patients, and among hospitals with respect to radiotherapy technology, segmentation plan and dose, and clinical target volume, which may have affected the results. It cannot be ruled out that a larger target volume of radiotherapy could increase the radiation damage to immune cells, leading to intensified immunosuppression. Second, the studies included in our analysis spanned 6 years, which may have affected the comprehensiveness of data collection. In the context of immunotherapy, there remain many unresolved questions regarding how to optimize a radiotherapy regimen. It is necessary to consider the best dose and fraction plan, treatment technology, and interval between radiotherapy and immunotherapy, as well as the impact of the clinical target volume. A focus on choice and safety is also important. A large number of clinical trials of immunotherapy combined with radiotherapy are currently underway. Circulating lymphocyte subsets have been shown to be biomarkers of immune status and to be related to the survival of multiple tumor types after radiotherapy. Other significant markers of immune status continue to be investigated, including humoral markers, cytokines, and tumor-infiltrating lymphocytes. These markers are often highly indication-specific and microenvironment-specific (58).

In every clinical setting that considers a combination of radiation (chemo) therapy and immunotherapy, it is very important to test the effect of RT on the immune system. It is worth noting that although changes in peripheral blood are easily monitored by routine blood sampling, they may not fully reflect the situation within the tumor, so these fast and low-cost blood biomarkers are used to identify the patient’s immune status and the impact of radiotherapy needs further research. The different immunomodulatory effects induced by radiotherapy may also depend on the heterogeneity of the tumor. In fact, tumor patients have complex biological heterogeneity. They are further classified into specific tumor subtypes and carried out in a larger patient cohort. The same analysis will better characterize the different immunomodulatory effects induced by radiotherapy, so that it may be possible to identify those patients who would benefit most from this potentially effective treatment.

## Conclusion

Lymphocytes are one of the most radiation-sensitive cell subgroups, accounting for about 30% of the total number of normal human white blood cells, and are essential effector cells in anti-tumor immunity (51).Changes in lymphocyte count are closely related to tumor progression and prognosis. As we all know, the changes of lymphocytes are related to the radiation source and the size of the radiation field. The analysis of the immune response at different time points may help to select the patients most likely to benefit from comprehensive treatment and prevent other patients from suffering unnecessary radiotherapy-related adverse effects. Therefore, our current research may pave the way for more effective cancer treatments (such as combined immunotherapy). Future work may benefit from the analysis of peripheral lymphocyte subsets at a large number of time points and the enrollment of more patients. However, the time change pattern after each radiation is not accurate. Peripheral sampling at other time points in a larger population may help clarify the time course of this phenomenon.

## Data Availability Statement

The datasets presented in this study can be found in online repositories. The names of the repository/repositories and accession number(s) can be found in the article/**Supplementary Material**.

## Author Contributions

XZ and QW conceived and designed this review. QW and SL conducted the literature search and collected the data. QW drafted the manuscript and figures. XZ, SL, SQ, ZZ, and XD reviewed and revised the manuscript. All authors contributed to the article and approved the submitted version.

## Funding

This work was supported by the National Natural Science Foundation of China [No. 81972853, No.81572279] and Clinical Research Startup Program of Southern Medical University by High-level University Construction Funding of Guangdong Provincial Department of Education (LC2019ZD009).

## Conflict of Interest

The authors declare that the research was conducted in the absence of any commercial or financial relationships that could be construed as a potential conflict of interest.

## References

[1] Shevtsov M Sato H Multhoff G Shibata A. Novel Approaches to Improve the Efficacy of Immuno-Radiotherapy. Front Oncol (2019) 9:156. 10.3389/fonc.2019.00156 30941308 PMC6433964

[2] Jarosz-Biej M Smolarczyk R Cichoń T Kułach N. Tumor Microenvironment as A “Game Changer” in Cancer Radiotherapy. Int J Mol Sci (2019) 20(13):3212. 10.3390/ijms20133212 PMC6650939 31261963

[3] Formenti SC Demaria S. Combining Radiotherapy and Cancer Immunotherapy: A Paradigm Shift. J Natl Cancer Inst (2013) 105(4):256–65. 10.1093/jnci/djs629 PMC3576324 23291374

[4] Ozpiskin OM Zhang L Li JJ. Immune Targets in the Tumor Microenvironment Treated by Radiotherapy. Theranostics (2019) 9(5):1215–31. 10.7150/thno.32648 PMC6401500 30867826

[5] Chajon E Castelli J Marsiglia H De Crevoisier R. The Synergistic Effect of Radiotherapy and Immunotherapy: A Promising But Not Simple Partnership. Crit Rev Oncol Hematol (2017) 111:124–32. 10.1016/j.critrevonc.2017.01.017 28259287

[6] Toulany M. Targeting DNA Double-Strand Break Repair Pathways to Improve Radiotherapy Response. Genes (Basel) (2019) 10(1):25. 10.3390/genes10010025 PMC6356315 30621219

[7] Baskar R Dai J Wenlong N Yeo R Yeoh K-W. Biological Response of Cancer Cells to Radiation Treatment. Front Mol Biosci (2014) 1:24. 10.3389/fmolb.2014.00024 25988165 PMC4429645

[8] Ma Y Pitt JM Li Q Yang H. The Renaissance of Anti-Neoplastic Immunity From Tumor Cell Demise. Immunol Rev (2017) 280(1):194–206. 10.1111/imr.12586 29027231

[9] McLaughlin M Patin EC Pedersen M Wilkins A Dillon MT Melcher AA. Inflammatory Microenvironment Remodelling by Tumour Cells After Radiotherapy. Nat Rev Cancer (2020) 20(4):203–17. 10.1038/s41568-020-0246-1 32161398

[10] Formenti SC Demaria S. Systemic Effects of Local Radiotherapy. Lancet Oncol (2009) 10(7):718–26. 10.1016/S1470-2045(09)70082-8 PMC2782943 19573801

[11] Demaria S Coleman CN Formenti SC. Radiotherapy: Changing the Game in Immunotherapy. Trends Cancer (2016) 2(6):286–94. 10.1016/j.trecan.2016.05.002 PMC5070800 27774519

[12] Demaria S Ng B Devitt ML Babb JS Kawashima N Liebes L. Ionizing Radiation Inhibition of Distant Untreated Tumors (Abscopal Effect) Is Immune Mediated. Int J Radiat Oncol Biol Phys (2004) 58(3):862–70. 10.1016/j.ijrobp.2003.09.012 14967443

[13] Lesueur P Chevalier F Stefan D Habrand J-L Lerouge D Gervais R. Review of the Mechanisms Involved in the Abscopal Effect and Future Directions With a Focus on Thymic Carcinoma. Tumori (2017) 103(3):217–22. 10.5301/tj.5000616 28291902

[14] Demaria S Golden EB Formenti SC. Role of Local Radiation Therapy in Cancer Immunotherapy. JAMA Oncol (2015) 1(9):1325–32. 10.1001/jamaoncol.2015.2756 26270858

[15] Blair TC Bambina S Alice AF Kramer GF Medler TR Baird JR. Dendritic Cell Maturation Defines Immunological Responsiveness of Tumors to Radiation Therapy. J Immunol (2020) 204(12):3416–24. 10.4049/jimmunol.2000194 PMC7571541 32341058

[16] Sheng H Huang Y Xiao Y Zhu Z Shen M Zhou P. ATR Inhibitor AZD6738 Enhances the Antitumor Activity of Radiotherapy and Immune Checkpoint Inhibitors by Potentiating the Tumor Immune Microenvironment in Hepatocellular Carcinoma. J Immunother Cancer (2020) 8(1):e000340. 10.1136/jitc-2019-000340 32461345 PMC7254123

[17] Arina A Beckett M Fernandez C Zheng W Pitroda S Chmura SJ. Tumor-Reprogrammed Resident T Cells Resist Radiation to Control Tumors. Nat Commun (2019) 10(1):3959. 10.1038/s41467-019-11906-2 31477729 PMC6718618

[18] Zhuang Y Yuan BY Chen GW Zhao XM Hu Y Zhu WC. Association Between Circulating Lymphocyte Populations and Outcome After Stereotactic Body Radiation Therapy in Patients With Hepatocellular Carcinoma. Front Oncol (2019) 9:896. 10.3389/fonc.2019.00896 31552194 PMC6748162

[19] Zheng Y Shi A Wang W Yu H Yu R Li D. Posttreatment Immune Parameters Predict Cancer Control and Pneumonitis in Stage I Non–Small-Cell Lung Cancer Patients Treated With Stereotactic Ablative Radiotherapy. Clin Lung Cancer (2018) 19(4):e399–404. 10.1016/j.cllc.2017.12.012 29519614

[20] Zhang C Dong J Shen T Li Y Yang Z Cheng X. [Comparison of the Application Among Intensity-Modulated Radiotherapy, 3D-Conformal Radiotherapy and Conventional Radiotherapy for Locally Advanced Middle-Low Rectal Cancer]. Zhonghua wei chang wai ke za zhi = Chin J Gastrointest Surg (2018) 21(12):1414–20. 10.3760/cma.j.issn.1671-0274.2018.12.015 30588595

[21] Yuan C Wang Q. Comparative Analysis of the Effect of Different Radiotherapy Regimes on Lymphocyte and Its Subpopulations in Breast Cancer Patients. Clin Transl Oncol (2018) 20(9):1219–25. 10.1007/s12094-018-1851-2 29536332

[22] Luo J Hu S Wei T Sun J Liu N Wang J. TGF-Beta 1 Levels Are Associated With Lymphocyte Percentages in Patients With Lung Cancer Treated With Radiation Therapy. OncoTargets Ther (2018) 11:8349–55. 10.2147/ott.s175956 PMC6267770 30568457

[23] Dovsak T Ihan A Didanovic V Kansky A Verdenik M Hren NI. Effect of Surgery and Radiotherapy on Complete Blood Count, Lymphocyte Subsets and Inflammatory Response in Patients With Advanced Oral Cancer. BMC Cancer (2018) 18(1):235. 10.1186/s12885-018-4136-9 29490633 PMC5831585

[24] Sage EK Schmid TE Geinitz H Gehrmann M Sedelmayr M Duma MN. Effects of Definitive and Salvage Radiotherapy on the Distribution of Lymphocyte Subpopulations in Prostate Cancer Patients. Strahlentherapie und Onkologie: Organ der Deutschen Rontgengesellschaft [et al] (2017) 193(8):648–55. 10.1007/s00066-017-1144-7 28500490

[25] Rutkowski J Slebioda T Kmiec Z Zaucha R. Changes in Systemic Immune Response After Stereotactic Ablative Radiotherapy. Preliminary Results of a Prospective Study in Patients With Early Lung Cancer. Polish Arch Internal Med (2017) 127(4):245–53. 10.20452/pamw.3997 28420863

[26] Sage EK Schmid TE Sedelmayr M Gehrmann M Geinitz H Duma MN. Comparative Analysis of the Effects of Radiotherapy Versus Radiotherapy After Adjuvant Chemotherapy on the Composition of Lymphocyte Subpopulations in Breast Cancer Patients. Radiother Oncol (2016) 118(1):176–80. 10.1016/j.radonc.2015.11.016 26683801

[27] Sangthawan D Phungrassami T Sinkitjarurnchai W. Effects of Zinc Sulfate Supplementation on Cell-Mediated Immune Response in Head and Neck Cancer Patients Treated With Radiation Therapy. Nutr Cancer (2015) 67(3):449–56. 10.1002/central/CN-01070880/full 25803777

[28] Chen Q Hu W Xiong H Ying S Ruan Y Wu B. Changes in Plasma EBV-DNA and Immune Status in Patients With Nasopharyngeal Carcinoma After Treatment With Intensity-Modulated Radiotherapy. Diagn Pathol (2019) 14(1):23. 10.1186/s13000-019-0798-0 30871579 PMC6417170

[29] Balazs K Kis E Badie C Bogdandi EN Candeias S Garcia LC. Radiotherapy-Induced Changes in the Systemic Immune and Inflammation Parameters of Head and Neck Cancer Patients. Cancers (2019) 11(9):1324. 10.3390/cancers11091324 PMC6770727 31500214

[30] Wild AT Herman JM Dholakia AS Moningi S Lu Y Rosati LM. Lymphocyte-Sparing Effect of Stereotactic Body Radiation Therapy in Patients With Unresectable Pancreatic Cancer. Int J Radiat Oncol Biol Phys (2016) 94(3):571–9. 10.1016/j.ijrobp.2015.11.026 PMC4847529 26867885

[31] Linares-Galiana I Berenguer-Frances MA Cañas-Cortés R Pujol-Canadell M Comas-Antón S Martínez E. Changes in Peripheral Immune Cells After Intraoperative Radiation Therapy in Low-Risk Breast Cancer. J Radiat Res (2021) 62(1):110–8. 10.1093/jrr/rraa083 PMC7779348 33006364

[32] Lv Y Song M Tian X Yv X Liang N Zhang J. Impact of Radiotherapy on Circulating Lymphocyte Subsets in Patients With Esophageal Cancer. Medicine (2020) 99(36):e20993. 10.1097/md.0000000000020993 32898991 PMC7478455

[33] Chen D Patel RR Verma V Ramapriyan R Barsoumian HB Cortez MA. Interaction Between Lymphopenia, Radiotherapy Technique, Dosimetry, and Survival Outcomes in Lung Cancer Patients Receiving Combined Immunotherapy and Radiotherapy. Radiother Oncol (2020) 150:114–20. 10.1016/j.radonc.2020.05.051 32525003

[34] Liu H Wang H Wu J Wang Y Zhao L Li G. Lymphocyte Nadir Predicts Tumor Response and Survival in Locally Advanced Rectal Cancer After Neoadjuvant Chemoradiotherapy: Immunologic Relevance. Radiother Oncol (2019) 131:52–9. 10.1016/j.radonc.2018.12.001 30773187

[35] Shibutani M Maeda K Nagahara H Fukuoka T Matsutani S Kashiwagi S. A Comparison of the Local Immune Status Between the Primary and Metastatic Tumor in Colorectal Cancer: A Retrospective Study. BMC Cancer (2018) 18(1):371. 10.1186/s12885-018-4276-y 29614981 PMC5883878

[36] Choi CH Kim WD Lee SJ Park W-Y. Clinical Predictive Factors of Pathologic Tumor Response After Preoperative Chemoradiotherapy in Rectal Cancer. Radiat Oncol J (2012) 30(3):99–107. 10.3857/roj.2012.30.3.99 23170288 PMC3496850

[37] Grossman SA Ellsworth S Campian J Wild AT Herman JM Laheru D. Survival in Patients With Severe Lymphopenia Following Treatment With Radiation and Chemotherapy for Newly Diagnosed Solid Tumors. J Natl Compr Canc Netw (2015) 13(10):1225–31. 10.6004/jnccn.2015.0151 PMC4778429 26483062

[38] Fang P Jiang W Davuluri R Xu C Krishnan S Mohan R. High Lymphocyte Count During Neoadjuvant Chemoradiotherapy Is Associated With Improved Pathologic Complete Response in Esophageal Cancer. Radiother Oncol (2018) 128(3):584–90. 10.1016/j.radonc.2018.02.025 29530432

[39] Joseph N Dovedi SJ Thompson C Lyons J Kennedy J Elliott T. Pre-Treatment Lymphocytopaenia Is an Adverse Prognostic Biomarker in Muscle-Invasive and Advanced Bladder Cancer. Ann Oncol (2016) 27(2):294–9. 10.1093/annonc/mdv546 26578732

[40] Sharma RA Plummer R Stock JK Greenhalgh TA Ataman O Kelly S. Clinical Development of New Drug-Radiotherapy Combinations. Nat Rev Clin Oncol (2016) 13(10):627–42. 10.1038/nrclinonc.2016.79 27245279

[41] Grossman SA Ye X Lesser G Sloan A Carraway H Desideri S. Immunosuppression in Patients With High-Grade Gliomas Treated With Radiation and Temozolomide. Clin Cancer Res (2011) 17(16):5473–80. 10.1158/1078-0432.CCR-11-0774 PMC3156964 21737504

[42] Bryant AK Mudgway R Huynh-Le M-P Simpson DR Mell LK Gupta S. Effect of CD4 Count on Treatment Toxicity and Tumor Recurrence in Human Immunodeficiency Virus-Positive Patients With Anal Cancer. Int J Radiat Oncol Biol Phys (2018) 100(2):478–85. 10.1016/j.ijrobp.2017.09.034 29102276

[43] Yang Z-R Zhao N Meng J Shi Z-L Li B-X Wu X-W. Peripheral Lymphocyte Subset Variation Predicts Prostate Cancer Carbon Ion Radiotherapy Outcomes. Oncotarget (2016) 7(18):26422–35. 10.18632/oncotarget.8389 PMC5041989 27029063

[44] Tang C Welsh JW de Groot P Massarelli E Chang JY Hess KR. Ipilimumab With Stereotactic Ablative Radiation Therapy: Phase I Results and Immunologic Correlates From Peripheral T Cells. Clin Cancer Res (2017) 23(6):1388–96. 10.1158/1078-0432.CCR-16-1432 PMC5355002 27649551

[45] Blum KS Pabst R. Lymphocyte Numbers and Subsets in the Human Blood. Do They Mirror the Situation in All Organs? Immunol Lett (2007) 108(1):45–51. 10.1016/j.imlet.2006.10.009 17129612

[46] Campian JL Piotrowski AF Ye X Hakim FT Rose J Yan X-Y. Serial Changes in Lymphocyte Subsets in Patients With Newly Diagnosed High Grade Astrocytomas Treated With Standard Radiation and Temozolomide. J Neurooncol (2017) 135(2):343–51. 10.1007/s11060-017-2580-z 28756593

[47] Ellsworth S Balmanoukian A Kos F Nirschl CJ Nirschl TR Grossman SA. Sustained CD4 T Cell-Driven Lymphopenia Without a Compensatory IL-7/IL-15 Response Among High-Grade Glioma Patients Treated With Radiation and Temozolomide. Oncoimmunology (2014) 3(1):e27357. 10.4161/onci.27357 24790790 PMC4004618

[48] Fadul CE Fisher JL Gui J Hampton TH Côté AL Ernstoff MS. Immune Modulation Effects of Concomitant Temozolomide and Radiation Therapy on Peripheral Blood Mononuclear Cells in Patients With Glioblastoma Multiforme. Neuro Oncol (2011) 13(4):393–400. 10.1093/neuonc/noq204 21339188 PMC3064696

[49] Crocenzi T Cottam B Newell P Wolf RF Hansen PD Hammill C. A Hypofractionated Radiation Regimen Avoids the Lymphopenia Associated With Neoadjuvant Chemoradiation Therapy of Borderline Resectable and Locally Advanced Pancreatic Adenocarcinoma. J Immunother Cancer (2016) 4:45. 10.1186/s40425-016-0149-6 27532020 PMC4986363

[50] McGee HM Daly ME Azghadi S Stewart SL Oesterich L Schlom J. Stereotactic Ablative Radiation Therapy Induces Systemic Differences in Peripheral Blood Immunophenotype Dependent on Irradiated Site. Int J Radiat Oncol Biol Phys (2018) 101(5):1259–70. 10.1016/j.ijrobp.2018.04.038 PMC6364565 29891204

[51] Mellman I Coukos G Dranoff G. Cancer Immunotherapy Comes of Age. Nature (2011) 480(7378):480–9. 10.1038/nature10673 PMC3967235 22193102

[52] Ménétrier-Caux C Ray-Coquard I Blay J-Y Caux C. Lymphopenia in Cancer Patients and Its Effects on Response to Immunotherapy: An Opportunity for Combination With Cytokines? J Immunother Cancer (2019) 7(1):85. 10.1186/s40425-019-0549-5 30922400 PMC6437964

[53] Cho Y Park S Byun HK Lee CG Cho J Hong MH. Impact of Treatment-Related Lymphopenia on Immunotherapy for Advanced Non-Small Cell Lung Cancer. Int J Radiat Oncol Biol Phys (2019) 105(5):1065–73. 10.1016/j.ijrobp.2019.08.047 31476418

[54] Venkatesulu BP Mallick S Lin SH Krishnan S. A Systematic Review of the Influence of Radiation-Induced Lymphopenia on Survival Outcomes in Solid Tumors. Crit Rev Oncol Hematol (2018) 123:42–51. 10.1016/j.critrevonc.2018.01.003 29482778

[55] Balmanoukian A Ye X Herman J Laheru D Grossman SA. The Association Between Treatment-Related Lymphopenia and Survival in Newly Diagnosed Patients With Resected Adenocarcinoma of the Pancreas. Cancer Invest (2012) 30(8):571–6. 10.3109/07357907.2012.700987 PMC3557506 22812722

[56] Burnette B Weichselbaum RR. Radiation as an Immune Modulator. Semin Radiat Oncol (2013) 23(4):273–80. 10.1016/j.semradonc.2013.05.009 24012341

[57] Eckert F Schaedle P Zips D Schmid-Horch B Rammensee H-G Gani C. Impact of Curative Radiotherapy on the Immune Status of Patients With Localized Prostate Cancer. Oncoimmunology (2018) 7(11):e1496881. 10.1080/2162402X.2018.1496881 30393582 PMC6208674

[58] Grassberger C Ellsworth SG Wilks MQ Keane FK Loeffler JS. Assessing the Interactions Between Radiotherapy and Antitumour Immunity. Nat Rev Clin Oncol (2019) 16(12):729–45. 10.1038/s41571-019-0238-9 PMC13492067 31243334

