# Supplementary material for: Changes in T Lymphocyte Subsets in Different Tumors Before and After Radiotherapy: A Meta-analysis
Source: Front Immunol. 2021 Jun 16;12:648652. doi: 10.3389/fimmu.2021.648652 (PMC8242248; doi:10.3389/fimmu.2021.648652)
Supplement: Supplementary file 2 [file DataSheet_2.docx]

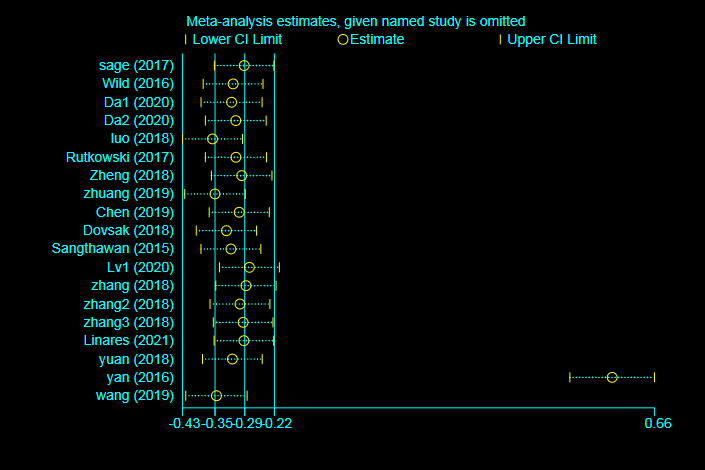


**Supplementary Material S2：Sensitivity analysis of CD3+ T lymphocyte changes after radiotherapy**


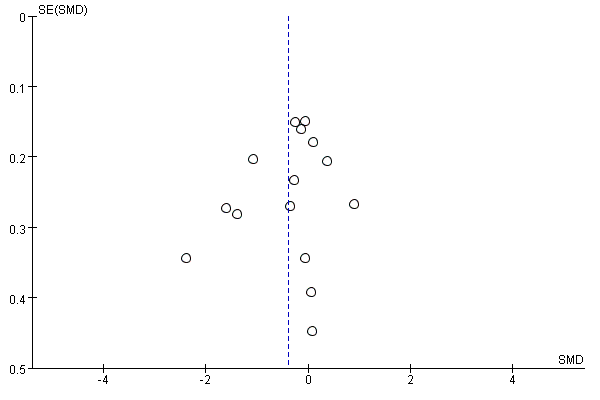


**Supplementary Material S3：funnel plot of CD3+ T lymphocyte changes after radiotherapy**

**
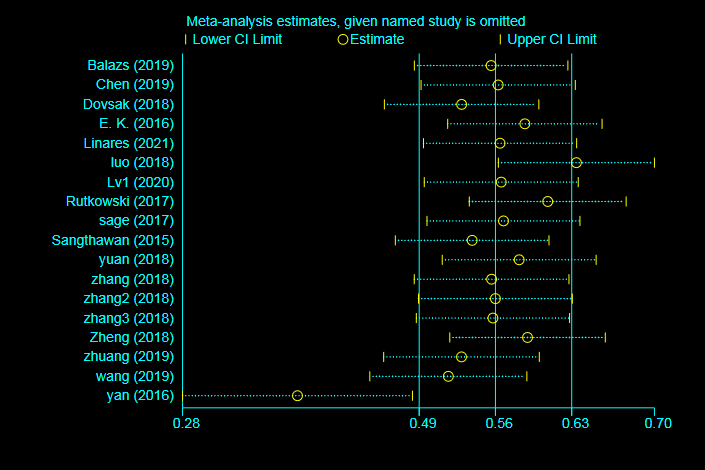
**

**Supplementary Material S4：Sensitivity analysis of CD4+ T lymphocyte changes after radiotherapy**

**
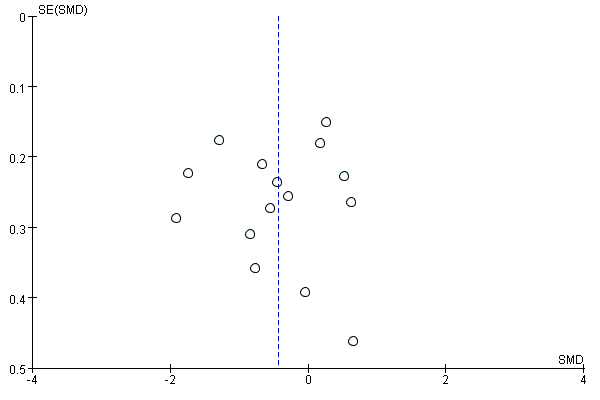
**

**Supplementary Material S5：funnel plot of CD4+ T lymphocyte changes after radiotherapy**


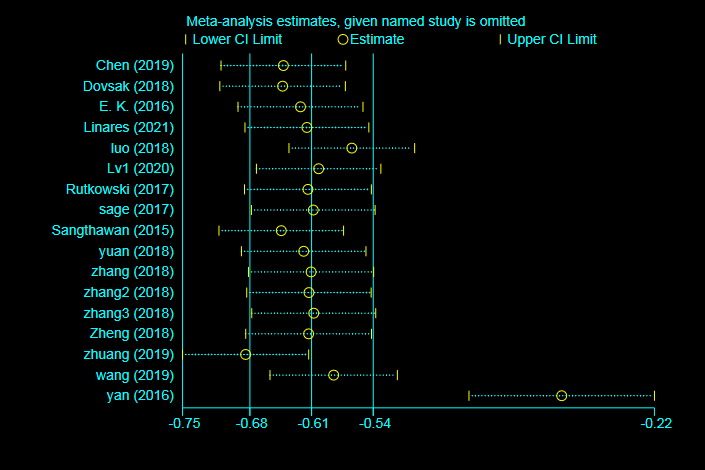


**Supplementary Material S6：Sensitivity analysis of CD8+ T lymphocyte changes after radiotherapy**


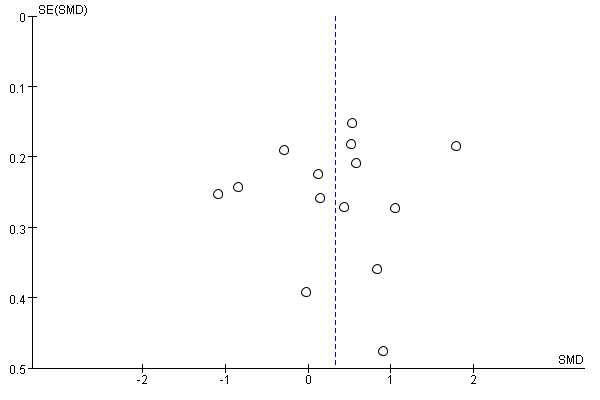


**Supplementary Material S7：funnel plot of CD8+ T lymphocyte changes after radiotherapy**

**
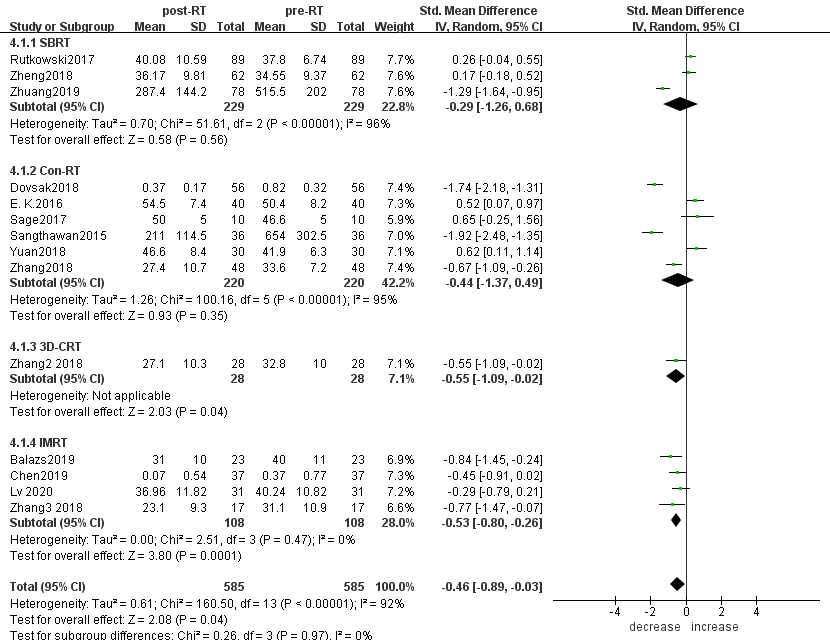
**

**Supplementary Material S8：Forest plots of subgroup analysis for changes of CD4+ T lymphocyte after radiotherapy compared with before radiotherapy with different radiotherapy technology.** Abbreviations: con-RT: conventional radiotherapy; SBRT: stereotactic radiotherapy ;IMRT: intensity-modulated radiotherapy ;3D-CRT： three-dimensional conformal radiotherapy

**
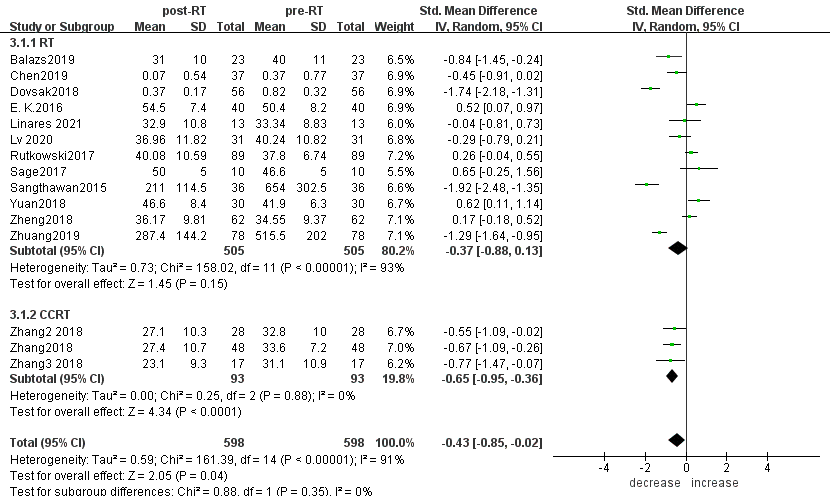
**

**Supplementary Material S9： Forest plots of subgroup analysis for changes of CD4+ T lymphocyte after radiotherapy compared with before radiotherapy in different treatment modes**


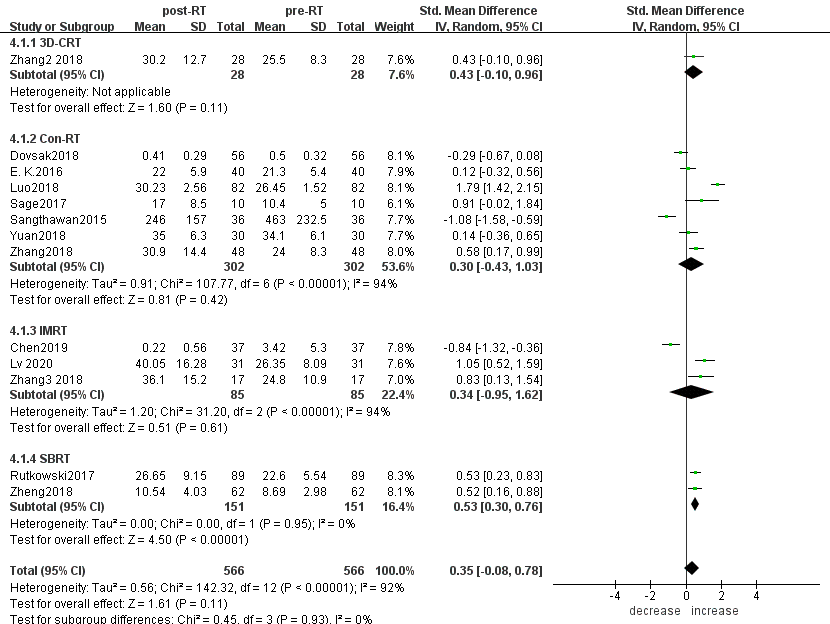


**Supplementary Material S10： Forest plots of subgroup analysis for changes of CD8+ T lymphocyte after radiotherapy compared with before radiotherapy with different radiotherapy technology.** Abbreviations: con-RT: conventional radiotherapy; SBRT: stereotactic radiotherapy ;IMRT: intensity-modulated radiotherapy ; 3D-CRT： three-dimensional conformal radiotherapy


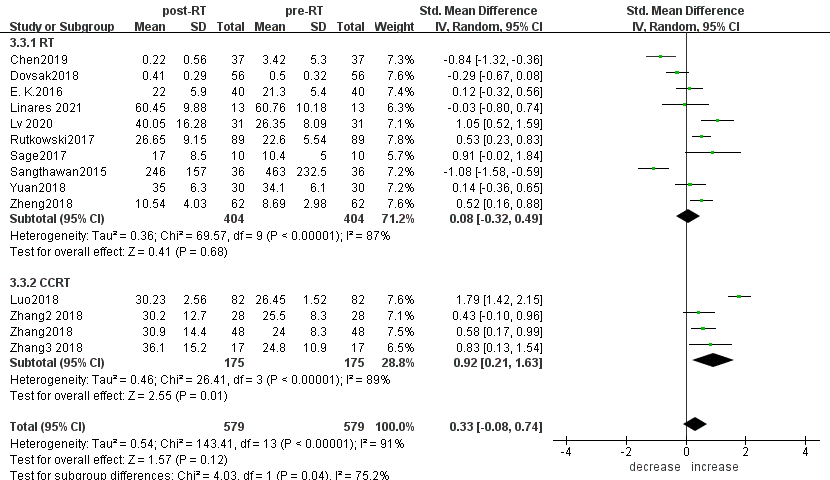


**Supplementary Material S11： Forest plots of subgroup analysis for changes of CD8+ T lymphocyte after radiotherapy compared with before radiotherapy in different treatment modes**

| **Author** | **Year** | **Sampe size** | **Age (years)** | **Country** | **Time to get the sample** | **RT  technology & dose** | **Cancer type** | **Post-RT VS Pre -RT** |
| --- | --- | --- | --- | --- | --- | --- | --- | --- |
| **[Lewin](https://pubmed.ncbi.nlm.nih.gov/?sort=date&term=Lewin+NL&cauthor_id=31519601) (1)** | **2019** | **93** | **65 median** | **Sweden** | **completion of RT VS. Pre-RT** | **adjuvant radiotherapy  50 Gy /25F** | **Breast Cancer** | **CD3(↓)、CD4+（↓）, CD8+（↓）** |
| **Gustafson(2)** | **2017** | **10** | **67.6 mean** | **United States** | **completion of RT VS. Pre-RT** | **SBRT 50 ~ 60Gy/5F, 54 Gy/3F** | **liver cancer** | **CD3（↓）、CD4+（↓）, CD8+（－）** |
| **Eckert(3)** | **2018** | **18** | **7****5 mean** | **Germany** | **1 month after RT VS. Pre-RT** | **RT 70–78 Gy/ 35–39F 50.4 Gy /28 F** | **prostate cancer** | **CD3（－）、CD4+, （－）、CD8+（↓）** |

1. Lewin NL, Luetragoon T, Shamoun L, Oliva D, Andersson BÅ, Löfgren S, et al. The influence of adjuvant radiotherapy and single nucleotide polymorphisms on circulating immune response cell numbers and phenotypes of patients with breast cancer. *Anticancer Research* (2019) 39(9):4957-63. doi: 10.21873/anticanres.13684.

2. Gustafson MP, Bornschlegl S, Park SS, Gastineau DA, Roberts LR, Dietz AB, et al. Comprehensive assessment of circulating immune cell populations in response to stereotactic body radiation therapy in patients with liver cancer. *Adv Radiat Oncol* (2017) 2(4):540-7. Epub 2017/12/06. doi: 10.1016/j.adro.2017.08.003.

3. Eckert F, Schaedle P, Zips D, Schmid-Horch B, Rammensee HG, Gani C, et al. Impact of curative radiotherapy on the immune status of patients with localized prostate cancer. *Oncoimmunology* (2018) 7(11):e1496881. Epub 2018/11/06. doi: 10.1080/2162402x.2018.1496881.

**Supplementary Material S12： 9 articles whose data could not be extracted and were excluded, among which 3 articles were qualitatively analyzed**
